# Educators’ experiences with governance in curriculum change processes; a qualitative study using rich pictures

**DOI:** 10.1007/s10459-021-10034-1

**Published:** 2021-03-01

**Authors:** Floor Velthuis, Hanke Dekker, Remco Coppoolse, Esther Helmich, Debbie Jaarsma

**Affiliations:** 1 Center for Education Development and Research in Health Professions (CEDAR), University Medical Center Groningen, Antonius Deusinglaan 1, 9713 AV Groningen, The Netherlands; 2 Research Group Normative Professionalization, University of Applied Sciences Utrecht, Utrecht, The Netherlands

**Keywords:** Undergraduate medical curriculum change, Governance, Implementation, Medical schools, Educators

## Abstract

In the midst of continuous health professions curriculum reforms, critical questions arise about the extent to which conceptual ideas are actually put into practice. Curricula are often not implemented as intended. An under-explored aspect that might play a role is governance. In light of major curriculum changes, we explored educators’ perspectives of the role of governance in the process of translating curriculum goals and concepts into institutionalized curriculum change at micro-level (teacher–student). In three Dutch medical schools, 19 educators with a dual role (teacher and coordinator) were interviewed between March and May 2018, using the rich pictures method. We employed qualitative content analysis with inductive coding. Data collection occurred concurrently with data analysis. Different governance processes were mentioned, each with its own effects on the curriculum and organizational responses. In Institute 1, participants described an unclear governance structure, resulting in implementation chaos in which an abstract educational concept could not be fully realized. In Institute 2, participants described a top–down and strict governance structure contributing to relatively successful implementation of the educational concept. However it also led to demotivation of educators, who started rebelling to recover their perceived loss of freedom. In Institute 3, participants described a relatively fragmentized process granting a lot of freedom, which contributed to contentment and motivation but did not fully produce the intended changes. Our paper empirically illustrates the importance of governance in curriculum change. To advance curriculum change processes and improve their desired outcomes it seems important to define and explicate both hard and soft governance processes.

## Introduction

Worldwide, medical schools seem to be in a constant state of reforming their health professions curricula. However, concerns are increasingly being raised about the extent to which conceptual ideas are actually put into practice and result in changes in the curriculum in action (Whitehead 2017; Hawick et al. 2017; Norman 2017). In health professions education literature, these concerns have been phrased as the curricular carousel, “*the recurrence of reforms with limited change*” (Whitehead 2017, p. 283), or “*the [continuous] model of endless epicycles*” (Norman 2017, p. 800). These phrases point to a major problem. Despite well-thought curriculum designs using educational concepts, interpretation and translation of these carefully crafted innovations to educational practice at micro-level (e.g. teacher–student) often does not meet the intended outcomes (Norman 2017). An under-explored aspect that might contribute to this mismatch is the role of governance.

Academic governance—in this paper understood as the means by which decisions are made, implemented and monitored in a curriculum—is a vital, yet rarely considered aspect of medical education practice (Casiro and Regehr 2018) As Casiro and Regehr argued, discussions about content and pedagogy should be complemented with discussions about governance frameworks capable of enabling curriculum change. “*Focusing on curricular changes and program evaluation while ignoring the processes of change (the mechanisms of decision making and implementation) is one of the key mistakes that lead to failed change efforts*” (Casiro and Regehr 2018, p. 2). A better understanding of governance processes related to curriculum change implementation might, therefore, improve our understanding of how to successfully translate educational concepts (e.g. a new educational philosophy or ideas for a new program such as problem-based learning) into actual changes in the curriculum in action.

Although the empirical literature on governance is scarce within the field of health professions education, there are some factors that should be considered. Some recent publications have addressed governance within academic medical centers (Guzick and Wilson 2018; Chari et al 2018; Pellegrini 2018), and higher educational institutes (Campbell and Bray 2018; Stensaker and Vabø 2013; Kezar 2004). Most of them focused on overall, institutional, macro-level governance, for example governance characteristics to ensure the success of the tripartite mission (patient care, medical education and research) of academic medical centers (Chari et al 2018; Pellegrini 2018) or ways in which universities describe shared governance in their strategic plans (Stensaker and Vabø 2013). However, governance processes on lower levels, such as the level of educators of medical curricula, remain understudied. This could be problematic as undergraduate medical curricula face unique governance challenges due to the complex interdependencies between universities and healthcare systems in which they are embedded. Additionally, there is a large variety of internal and external stakeholders who feel that they have a stake in the process and the ‘final product’ (Casiro and Regehr 2018). At curriculum level, complex interactions exist between a large number of staff, educators, course coordinators and curriculum leaders (Casiro and Regehr 2018; Velthuis et al. 2018). Furthermore, governance processes often remain implicit as discussions tend to focus on curriculum content and design rather than explicating decision making practices (Casiro and Regehr 2018). This is particularly problematic as medical curricula constantly need to respond to change (e.g. changing needs of society, healthcare, their own healthcare institution and education), which makes them dynamic, evolving, high-stake programs. Therefore, it is essential that medical schools carefully consider these organizational factors to function effectively and ensure effective curriculum delivery (Casiro and Regehr 2018). More empirical research is needed to inform effective academic governance at this lower organizational level.

What we already know about good (also referred to as effective) governance is that it encompasses hard and soft aspects (Birnbaum 2004). ‘Hard’ aspects relate to formal rules, procedures and processes that form the framework of governance of an institution (Chari et al 2018), and include elements like committee structures, lines of reporting, and clarity about responsibility, accountability and authority (Casiro and Regehr 2018). Within this framework, various ‘soft’ human processes further shape the governance system’s functioning. This concerns leadership and trust (Kezar 2004), relationships, participation, communication, perceived fairness and transparency and legitimacy of the decision making processes by those involved (Casiro and Regehr 2018). However, empirical studies on how these governance processes are perceived by i.e. staff members who are closely involved in change processes and shape the implementation, are lacking.

In undergraduate medical education, educators operate at this lower level and fulfill a key role in translating new curriculum ideas into practice. Although they play a vital role in curriculum enactment, little is known about how they experience governance processes in curriculum change. Knowing more about educators’ perceived struggles and their experiences with governance will help us better understand how decisions are made and implemented. A deeper understanding of difficulties and challenges that are faced can help identify deficiencies and needs for support which, in turn, will enhance curriculum governance practices. To bridge the gap, this paper explores how educators perceive the role of governance in the process of translating curriculum goals and concepts into institutionalized curriculum change at micro-level (teacher–student).

## Methods

The Netherlands Association for Medical Education ethical review board approved this study (number: 965).

### Context

In the Netherlands, undergraduate medical education is provided by 8 medical schools. Dutch curricula comprise a 3-year Bachelor’s and a 3-year Master’s phase. Three medical schools that had implemented a new educational concept/philosophy resulting in a major curriculum change, were invited to participate in our study. We defined major curriculum change as: “*changes that [are] not about the yearly, regular adjustments at course level, but [are] centrally organized, intentionally initiated change projects that affect the entire curriculum and organization involved in the curriculum*” (Velthuis et al. 2018). These changes had been made in the Bachelor’s phase of the curriculum. In this phase, the focus is predominantly on gaining basic science and medical knowledge and developing competencies following the CanMEDs model. Usually, in this phase, students have their first patient encounters. In the predominantly clinically oriented Master’s phase, the number and variety of patient encounters increases. Each school admits an average of 400 students per year.

### Design

In each medical school, we carried out interviews with 5–7 educators. We used Rich Pictures, a drawing tool from systems engineering, to augment data collection (Cristancho 2015; Checkland 2000). The rich pictures method is useful in describing and understanding messy or complex problems, as the pictures aim to reflect on a ‘reality’, with all its interacting elements such as people, objects, emotions, beliefs and relationships (Cristancho 2015). Rich pictures capture these interplays in one drawing and provide insights into the multiple interactions taking place simultaneously (Checkland 2000). Since we wanted to explore the complexity of curriculum change governance, this method was appropriate.

Our study was conducted from a constructivist orientation, as we believe that knowledge is co-constructed between participants and researchers and that multiple realities occur in social processes (Guba and Lincoln 1994), like curriculum change. These realities are constructed in the curriculum change context, but also (re)constructed during drawing and the interviews. As a team, we further co-constructed the interpretations of our analysis during frequent team discussions. Therefore, our backgrounds also serve as an important factor, shaping our results: FV studied social psychology and is interested in organizational processes of curriculum change. HD is a senior educationalist chairing educational development task-groups and RC is a senior curriculum innovation consultant. The latter two are dealing with curriculum (change) matters such as governance on a daily basis. AJ is a professor in health professions education, who has experience with curriculum changes in several institutes. Finally, EH is an elderly care physician and an experienced rich picture researcher. When we started this study, none of the authors was involved in the curriculum change processes of the three medical schools.

### Recruitment

The deans of education were asked for permission to conduct research in their medical school. FV sent them an information letter by email, explaining the purpose of the study and requirements for participation. The target group consisted of the bachelor curriculum coordinator and educators who were both teaching and coordinating a course in the curriculum, and willing to share their experiences with the governance processes related to the most recent curriculum change. Subsequently, the deans of education introduced the study by distributing the information letter among potential participants as they saw fit. Those who were interested in participating were invited to contact FV.

### Participants

All participants (7 female, 12 male) had been teaching for many years within medical education and worked as basic scientists or physicians. Some had participated in the preparatory phase of the curriculum change process, others were primarily involved in the implementation phase of translating the ideas into actual curriculum practice. They all had a dual role as teacher in the curriculum and as course coordinator, so they were all involved in providing education, developing educational materials and evaluating the curriculum. Apart from teaching, the bachelor curriculum coordinators (one in each institute), fulfilled a different role. They were responsible for the content, quality assurance and coherence of the entire bachelor curriculum at a more executive, daily level.

In two cases, the curriculum change processes the participants looked back at started around 3 years before the interviews, and the implementation of year two and three of the program was taking place. In another institute, the change process happened 5 years before, and each of the 3 years of the undergraduate program had been taking place for at least 1 year.

### Data collection

Between March and May 2018, each school was visited for a few days in a row to conduct the interviews. FV conducted 15 individual face-to-face interviews and, for feasibility reasons and after getting instructions and practicing together with FV, RC conducted the remaining 4 interviews. FV studied the literature on academic governance and developed the interview guide in frequent consultation with the team. To refine the interview guide, FV conducted two pilot interviews with a curriculum coordinator and an educator outside the target population.

The term governance is not often used in Dutch and also doesn’t have a translation. Therefore, one week prior to the interviews, to help participants understand what we wanted to talk about, we send them more information about the topic. We used Casiro and Regeher’s definition of governance: ‘*the means by which decisions are made, implemented and monitored in a curriculum*’ (Casiro and Regehr 2018). Furthermore, we explained that we were interested in both hard (e.g. structures, lines of reporting, responsibilities, accountability) and soft aspects (e.g. perceived fairness, legitimacy, communication, relationships) of governance (Casiro and Regehr 2018; Kezar 2004; Birnbaum 2004). Additionally, to help participants in narrowing a broad topic as governance, we first asked them to think about a case to illustrate the governance processes in their medical school’s curriculum change implementation process. During the interviews, the discussions took a broader perspective, and often involved participants’ perceptions about the entire governance processes in relation to their institute's curriculum changes.

We started all interviews repeating the above-mentioned explanation of governance. Hereafter, participants were asked to draw the governance case they had in mind and to include all aspects that mattered to them in the rich picture (buildings, people, objects, relationships, emotions, tensions, et cetera) (Cristancho 2015). Each participant was granted half an hour to draw the case and left alone with a large white paper and color pencils. Subsequently, a semi-structured interview was held, in which the rich picture served as a mean for participants to describe their governance experiences. In addition to explorative questions about the drawing and its meaning, challenging questions were asked like: what are consequences of the described governance processes for educational outcomes/educational practice? How do the processes you describe help or hinder the implementation/translation process? What is your opinion about the final result; was the intended change actually realized in practice? In total, sessions lasted between 1 and 1.5 h. The drawings were photographed, and interviews were audio-recorded and rendered anonymous in the transcription process.

### Data analysis

We employed qualitative content analysis, a “*dynamic form of analysis of verbal and visual data that is oriented towards summarizing the informational contents of that data*” (Sandelowski 2000, p. 338). Qualitative content analysis is an inductive coding process, with data collection occurring concurrently with data analysis. The result is a descriptive summary of the event of study (Sandelowski 2000).

After each interview round in a medical school, FV showed the pictures and introduced the interview contents to AJ, EH and HD. In this stage, illustrative figures and metaphors in the drawings were identified and, when appropriate, selected to visually enrich the results section of our paper. To this end, the selected drawings were anonymized and (if applicable) the Dutch words were translated into English. Concerning the analysis of the interview data, FV started with detailed, open coding of five interviews using Atlas.ti 8 (ATLAS.ti Scientific Software Development GmbH, Berlin), followed by discussions with EH and AJ who shared their reflections on these interviews. After consensus was reached about the coding, FV moved into more focused coding of all interviews from one institute and wrote summaries of each interview to grasp the main messages. To combine all the interviews of one institute, FV used the Atlas.ti network-tool to create an overview of the codes and their connections. Subsequently, FV discussed the developing network with EH, HD and AJ to ensure accuracy and reach agreement about the interpretations. This process was repeated for the other two institutes (coding, summarizing, creating networks). Regular team meetings were held to discuss the process and the team’s evolving interpretations. Member checking was used to validate the participants’ responses; all participants agreed with the way the results were presented.

## Results

**Fig. 1:**
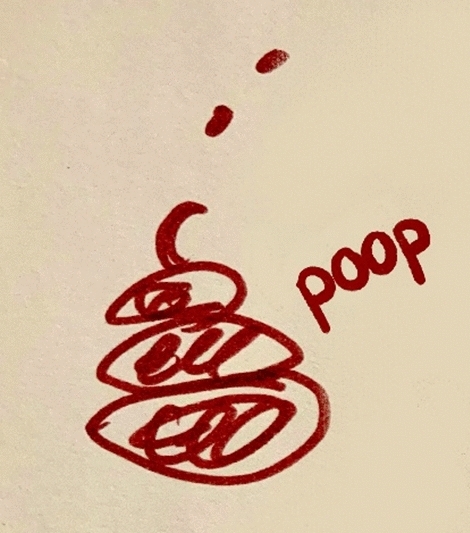
Decision making drawn and explained as ‘a great pile of shit.’ (P11)

**Fig. 2:**
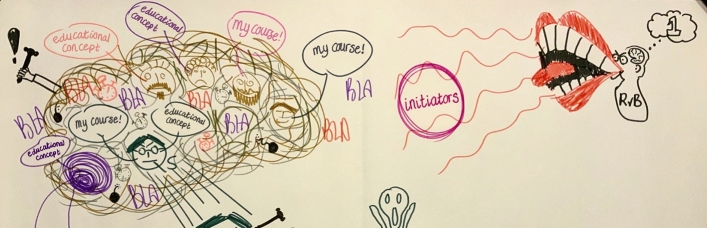
Having the Board of Directors on the right, represented as screaming with a big mouth to the curriculum committee members about what should be done, while dreaming to be number one in the rankings, and having little brains and small ears, expressing that they were not perceived to have the brains to know what should be done, and being bad listeners. On the left the representation of implementation chaos in the curriculum committee as a dirty plate of spaghetti. (P9)

**Fig. 3:**
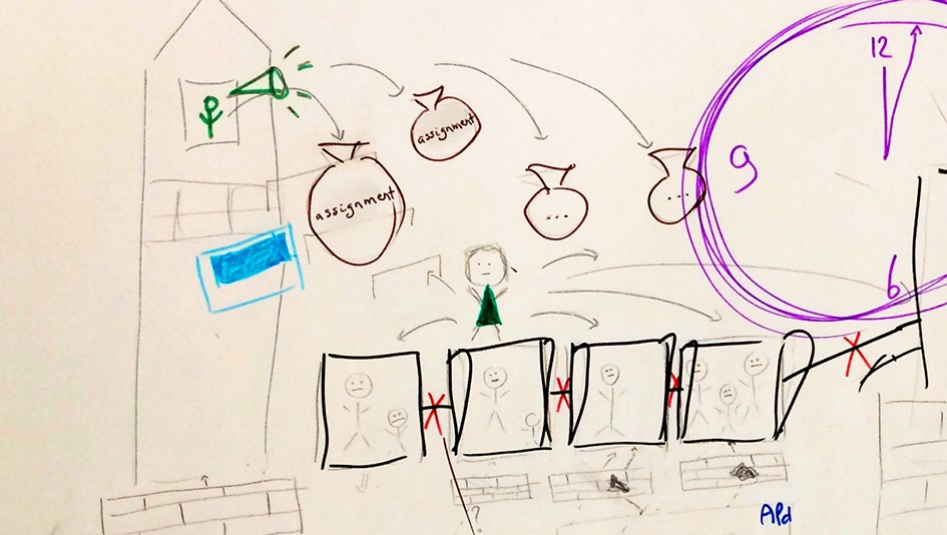
In the tower of the educational institute, the head of the curriculum is screaming and throwing bags, which are represented to be the assignments for the organization about what they should do regarding the development of the new curriculum. The person receiving the bags is not happy, as is expressed by the sad face, and is trying to covey the messages to others with whom he/she is working but that does not work, as everybody is already overloaded and too busy with their own parts, represented as the wall that stand between them with red crosses, representing no connection. (P8)

**Fig. 4:**
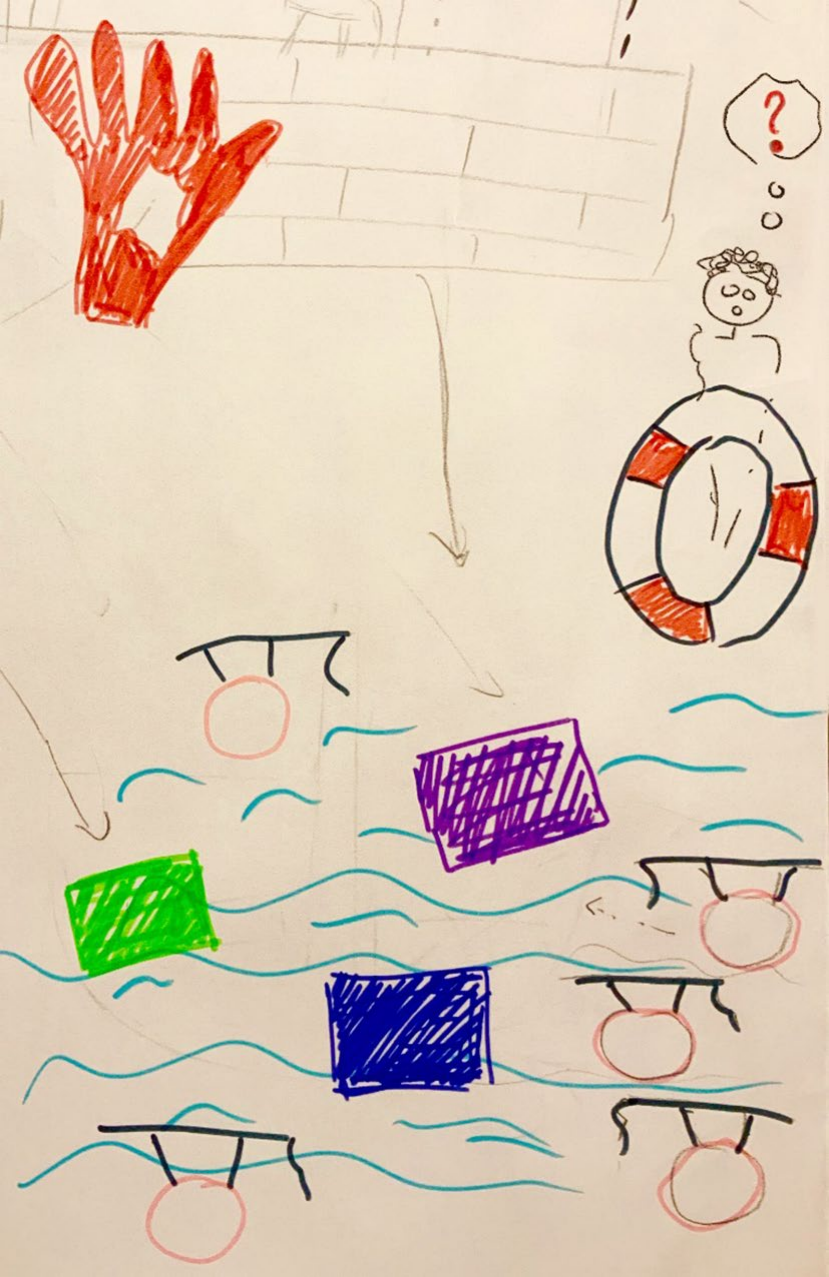
Below, students are represented to be swimming between different educational materials/tools (the blocks), not knowing where to go to, with a teacher who wants to help them, however is also uncertain him/herself about what to do and what is expected of this new curriculum. The big red hand on the left represents a stop sign of the department that was responsible for the logistics and scheduling of the new curriculum. They were explained to be at some point so entirely overloaded (outside this snapshot; people crying really hard) that they were not approachable anymore, having negative consequences for the continuation and implementation of the change process. (P8). (Color fiogure online)

For clarity, each of the three institutes will be addressed separately by describing and visually illustrating participants’ experiences with and perceptions of governance processes related to their own approaches to implement curriculum changes, and how these processes affected whether the desired changes were met in practice or not (Figs. 1, 2, 3 and 4).

### Institute 1

**Fig. 5:**
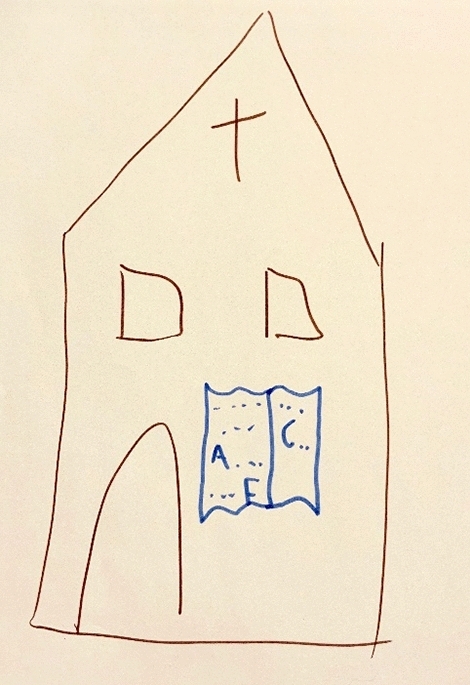
the Blueprint represented as Bible in a church. (P5)

**Fig. 6:**
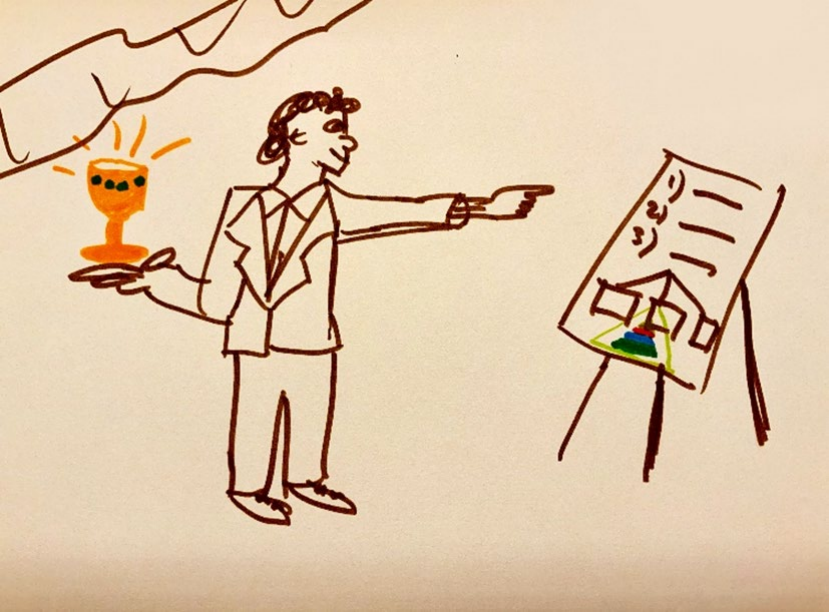
The educational scientist represented with the holy grail in his left hand, pointing at the outlines of the curriculum on the board on the right. (P2)

In Institute 1, one central educational concept had to be implemented together with achievement of goals aiming for more coherency between educational courses, better alignment between teachers, and the integration of clinical and preclinical subjects. Implementing these goals proved to be somewhat problematic. To illustrate this, a participant drew a “*huge pile of shit*” to explain the governance processes (Fig. 5, P11), and another participant used the “*ugliest colors*” to draw a “*dirty plate of spaghetti*” representing the chaos of decision making (Fig. 6, P9). These metaphors illustrated an ambitious, yet frustrating and chaotic curriculum change process, in which clear decision making was lacking as perceived by participants. This, in turn, caused troubles that persisted throughout the entire change process according to them.

**Fig. 7:**
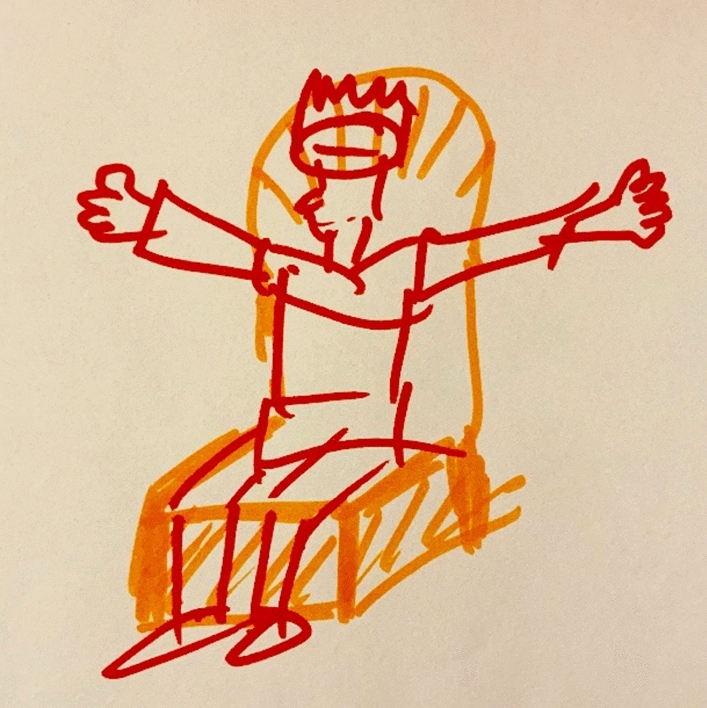
The curriculum director represented as a king on the thrown. (P2)

As participants explained, the idea was to radically change the curriculum. According to those who had been involved in earlier stages of the curriculum change, the starting point was an outline on paper about the new educational concept, which was considered to be too philosophical and, therefore, vague and abstract. The lack of time and the change leaders’ strong focus on progression offered little room for more in-depth discussions to establish a common understanding of the new educational concept and what it actually meant in practice (Fig. 7). As one described:

No time was allotted to develop a shared vision of how we are going to translate this [educational concept]. (P9).

Additionally, participants perceived the curriculum committee’s discussions as inefficient, since no official decisions were made, resulting in repeated discussions about issues that had already been decided upon. This created ambiguity, which was perceived to be mainly caused by a lack of clarity about who was ultimately responsible for decision making and ambiguity about roles, tasks, authority and leadership.

This perceived lack of clear decision making resulted in what participants referred to as “*implementation chaos*” (*P9*), which created more time pressure. The participants experienced the governance processes to be very top–down. One of the participants described and illustrated this as heavy bags thrown from the tower, and explained:

One man is shouting from the top of the tower and throwing down (…) the assignment into the organization while providing some background information: “you have to develop the new curriculum and make sure that it works.” This was done under very high time pressure. According to me, we knew half a year before [the new curriculum would start] what we actually had to do, so that felt like all of a sudden a heavy bag was falling down on you. (P8).

Furthermore, participants were unsatisfied with decisions made about the education support structures in their medical school. They experienced that both educational scientists of the faculty development department and faculty of the scheduling/logistics department were not optimally involved in the process and, therefore, felt insufficiently supported in their efforts to adapt to the major changes. Consequently, they were insufficiently able to support the major changes themselves. This was said to hinder effective implementation of the radical changes. In addition, the lack of time and, therefore, the high time pressure, was explained to result in teachers predominantly focusing on their own work rather than working together. This did not support the desired integration of subjects in the curriculum. Overall, participants experienced many teething troubles in the first years. They felt that students were suffering from the chaotic situation, illustrated by one participant, drawing students swimming around a variety of educational materials and tools, without any cohesion or structure, and a teacher with a lifebuoy, trying to save them however, also unsure him/herself about what to do, and what is expected. To overcome these shortcomings, formal evaluations by students were mentioned to be an important factor in decision making processes in curriculum adjustment.

Looking back, participants thought that the disruptive approach had supported in shaking things up to create something new and different. However, the final result was not satisfying. The medical school’s governance processes did not support clarification of the meaning of the goals of this new educational concept. As this affected the entire implementation process, it was explained that teachers ended up in making their own interpretations. This resulted in a large variation of educational methods and translations of the concept which, in turn, caused uncertainty and ambiguity among both teachers and students. As a participant looked back:

Learning from that [period], I now think: at least governance needs to be clear before we start working on something. (P11).

After the curriculum change was implemented, a new governance structure was established. Within this new structure, several stakeholders were involved in the discussions about curriculum adjustments, but the final decisions were made by one curriculum coordinator, who was ultimately responsible for the entire curriculum. Participants believed that this new structure had greatly clarified the decision-making process.

### Institute 2

In this institute, also one central educational concept was chosen to be implemented. Additional goals were to create more coherence between courses and integration of clinical and preclinical subjects. In Dutch medical schools, a document outlining the new curriculum on paper is usually referred to as ‘the curriculum blueprint’. In our study in institute 2, the blueprint turned out to be the core element of the drawings and interviews about governance in this medical school. The blueprint was broadly applied as central governance instrument in formal decision making. A metaphor of the Bible represented this formal, clear, strict and centrally managed structure through which curriculum decisions were explained to be made.

So, this is my Bible, so to say. And similar to the Bible you can question some stories, however, this just happens to be the principle/the start of the Creed that is called our new curriculum. The Blueprint decreed, therefore, that the chosen educational concept should play a central role in our curriculum. (P5).

A formally constituted curriculum committee monitored compliance with the blueprint during curriculum implementation and recalled course coordinators and teachers when they deviated from the guidelines:

There are also a few educationalists [in this committee] who, every time that too much freedom is created, by me, or by teachers, push me back to the blueprint. (P5).

The centrally organized governance structure was also established to increase cohesion while avoiding so-called ‘islands’ that occurred in the previous curriculum, on which teachers worked too much in isolation. As a participant explained:

This [centrally organized governance structure] was also deliberately chosen, and this was also announced in advance: we have to go to a more centrally organized structure, in which course coordinators cannot play the king over their own educational course anymore. (…) In the past, that was a process that got out of hand (…). Where did the consistency remain in the curriculum? In that sense, I do understand [this approach], (…) but it also creates tensions, of course. (P1).

These tensions related to participants experiencing the process to be top–down leading to feelings of being restricted as an educator by the rules of the curriculum committee:

I fail to achieve my own objectives in adhering to the holy grail of educational scientists. And ultimately, if I don’t adhere [to it], the King [the curriculum coordinator] says: listen to the holy-, he knows how it works, do what he says! (P2).

**Fig. 8:**
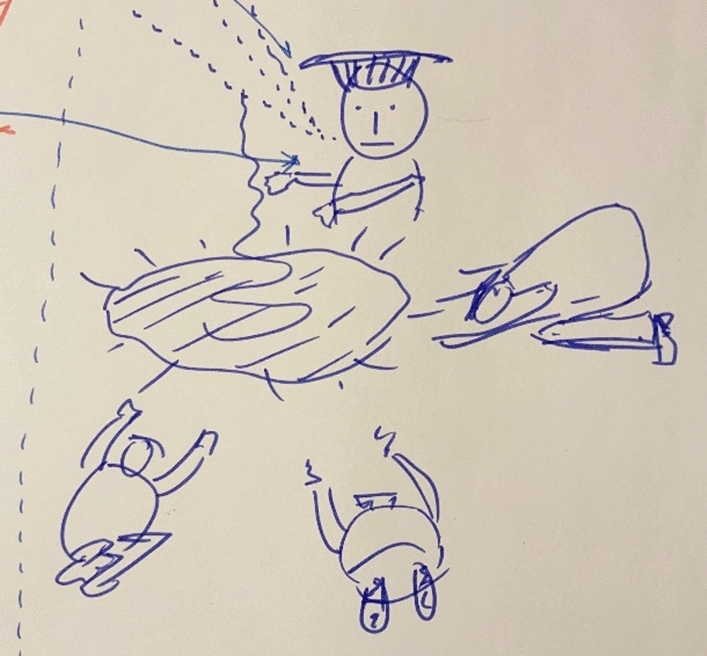
The representation of people worshipping the external Oracle that was listened to by curriculum leaders instead of to the ideas of faculty. (P1)

Another example of tensions related to the way decisions were made, was a lack of trust in curriculum leaders, who were explained to be predominantly listening to an external expert in medical education—metaphorically drawn by a participant as people worshipping the oracle—instead of listening to faculty’s ideas or suggestions; see Fig. 8.

In the strict governance structure, participants experienced a strong loss of freedom and autonomy regarding their classroom practices as well as their participation in the change process. The blueprint dictated the teaching approach they had to take in the new curriculum with regards to content and pedagogy. Furthermore, a repeated complaint was that only a small group of people was involved in decision making about the new curriculum. Teachers experienced that they were not given enough opportunity to participate in the curriculum development process, think along pro-actively, or have a voice in it. For the participants, this led to feelings of not being acknowledged and valued for their expertise.

In response to this strict approach, participants found ways to deviate, out of sight, from the blueprint. They sought freedom/space to pave their own path, because the new methods looked good on paper but did not work well enough in practice. They experienced a mismatch between the dictated educational methods and the content of their teaching, tensions between more time-consuming methods and the amount of study materials that had to be taught within the already limited time available (the risk of curriculum bloating), and felt that it was sometimes artificial to integrate teaching subjects. As a result, participants created workarounds:

We discuss this with the other coordinators in our course (…) but of course we are not going to mention all these small changes [to the head of the curriculum] (P6).

Deviations from the guidelines, in turn, created tensions amongst participants. For example, some participants seemed to interpret others’ resistance as unwillingness to change or failure to take the change seriously:

For a strikingly long period of time people are allowed to disagree (…) According to me, too structurally, there are people who think like: ‘okay, the new curriculum, I’ll continue with the old one and put a dollop of whipped cream on top, that’ll be enough’. I think this is pretty much tolerated in general. (P4).

In general, the so-described top down, centralized and strict structures and procedures with the blueprint as main instrument, were perceived to be relatively supportive of getting the new educational concept off the ground. However, the participants experienced that the governance structure was too top-down, which resulted in a lack of support for decisions that were made by only a select group of people. Educators’ lack of autonomy in decision making created tensions, causing them look for workarounds as a way of compensating their loss of freedom. All in all, these divergent responses threatened the internal cohesion of the curriculum as well as educators’ motivation, willingness to change and enthusiasm to be part of the curriculum development process.

### Institute 3

The overall goal of this curriculum change was to stimulate students’ active learning by using several methods. Additionally, participants explained that it strived for more cohesion, integration of knowledge and competencies, and integration of clinical and preclinical subjects. The participants described a new, yet developing, more centralized governance structure in which many people were still seeking thorough understanding of their own and others’ roles and responsibilities and the structure and roles within committees.

Considering the new curriculum, participants described a variety of methods that were used, based on different educational philosophies and principles. As a participant summarized:

We adopt a number of [educational] aspects and it still feels a little fragmented, different themes, it is not one structure, like ‘we work according to [the principles of] problem based learning’ for example, or ‘we employ team-based-learning, and that is how we design our curriculum’. The advantage is that you can do your own thing, which is nice, however, sometimes it’s a disadvantage. Then I wonder: To what extent is this clear to students? (P18).

A curriculum committee had developed the outlines of the new curriculum. However, just before the actual implementation the committee was dissolved. Consequently, only a few people were having a detailed overview of the entire new curriculum. This decision was mentioned as a shortcoming that affected the solidity of the new curriculum and remained problematic for faculty who had to develop and implement the new curriculum:

[The curriculum committee members] were the only ones having an overview, also in detail, of what happened where [in the new curriculum]. (…) It is still very difficult to get an overview of what is happening where. (…) The fundament, the solid base of the curriculum, its source, was in the committee, which was dissolved. (P15).

Together with the new curriculum, also a new governance structure was adopted to ease decision-making and a new educational management was installed. Participants were happy that the managers were trying to bring more coherency. As a participant explained:

I think it’s good that this [new educational management] is there because otherwise there would be no decision making at all. The only decisions that would be made, would be my own decisions about the course. And now, the [new educational management] monitors, I assume, the whole [curriculum]. I assume they try to create unity. (P13).

However, the new structure did not seem to be fully mature at the time of the interview. Although it was explicitly mentioned that the institute strived for more centralized decision making, many decisions and outcomes of the new curriculum still were described to be predominantly based on personal initiatives and understanding. Faculty autonomy is highly valued. As a participant described:

Actually, it’s up to [faculty] to decide how the Raamplan^1^ is going to be transposed [into the curriculum], to a certain extent. (P14).

Indeed, participants experienced a lot of freedom to pave their own path and determine what their courses will look like. Although freedom and autonomy were highly appreciated, they also threatened the aspired alignment and cohesion of the curriculum. Participants described how they predominantly worked on their own educational course or observed others doing that, without connecting to each other. Integration of subjects, therefore, appeared to be depending on each other’s willingness to collaborate and integrate in many situations. Consequently, the educational outcomes varied and were depending on internal (personal) relationships. A participant explained how decisions about involvement in other courses were taken:

That depends on the relationships with other course coordinators, and whether they have asked for it or have indicated to appreciate it. The course coordinator compiles that [group]. I think that [the group composition is based on what] he thinks is important for his course. (…) We have always actively approached the course coordinator to make sure we had [enough] time in the new curriculum. (P17).

Also in this institute, students’ input played an important role in decision making about curriculum adjustments. Participants experienced a clear evaluation structure with specific methods to evaluate the curriculum with students. However, some participants believed that nothing or very little was done with the results:

There is an evaluation meeting between student representatives and the year coordinator. (…) However, afterwards, nothing is done with the evaluation report. I’ll extract some things [i.e. information] that I can use, but nothing else is done with it. (P13).

The governance structure fostered the autonomy of faculty, which was perceived to be highly valued by them and to positively affect their motivation and happiness. However, it did not seem to be perceived as successful in bringing about the desired curriculum changes. The integration of subjects and implementation of teaching approaches to promote active learning did not reach the desired level and some participants doubted whether the coherence of the program was clear to students. Many activities were described to be based on individual initiatives and at one’s own discretion. Whether things or ideas were actually implemented seemed to depend on individual initiatives rather than central coordination. Continuous efforts to keep improving central decision making were claimed to be made. As a participant articulated:

It is all very informal. (…) For the next curriculum change we cannot work like this, we need to work more formal. (P14).

## Discussion

In this study, we explored how educators experience governance in curriculum change processes. We focused on the processes of translating curriculum goals and educational concepts on paper into actual curriculum practices. Our findings suggest that the way decisions are made, implemented and monitored (which is the definition of governance we used in this paper) plays a prominent role in the process of development and enactment of a new curriculum. The participating institutes seemed to have different governance processes, each with their own consequences for the curriculum and the way educatores enacted the intended practices and developed their courses. All processes reflected interactions between ‘hard’ (e.g. procedures, authority, responsibilities) and ‘soft’ aspects of governance (e.g. the perceived fairness, trust, legitimacy, relationships) (Birnbaum 2004). For example, participants of Institute 2 emphasized the ‘hard’ governance aspects with the blueprint as central governance instrument and centrally managed structures around it, like the curriculum committee controlling the people and processes. However, educators’ involvement in decision making and lack of confidence in the decisions made—which are examples of ‘soft’ governance aspects—were perceived as not being considered well enough and, therefore, some educators disagreed and looked for workarounds to reclaim their freedom and autonomy. This was deemed to pose a threat to achieving the curriculum change goals, such as institutional and curricular cohesion, and to negatively impact educators’ motivation. In the other two institutes, a clear governance procedure was less apparent or experienced to be lacking, albeit on different levels. In Institute 1, we recognized similarities in participants’ experiences of imposed change, described as a top-down process with a lack of attention for faculty involvement in decision making. In addition, a lack of clarity about leadership, authority, tasks and roles led to frustrations about the process and dissatisfaction with what the curriculum looked like in practice. In Institute 3, the more loose, teacher-centered governance structure almost showed the opposite. Here, the soft aspects of governance, such as faculty’s perceived decision freedom in the marginally centralized curriculum and their experienced impact of internal relationships on decision making processes and curriculum enactment, caused feelings of contentment and happiness. However, the same arguments were put forward to explain a lack of structural collaborations to establish curriculum integration and coherence of the new curriculum. This illustrates the importance of balancing hard and soft governance aspects in determining “the right way of doing things”, which was perceived as challenging and highly context dependent.

Our study empirically illustrates many of the governance aspects that Casiro and Regehr described in their paper (Casiro and Regehr 2018). An aspect that was particularly striking to us was that people were circumventing the system. Educators disagreed with the governances processes, which were not always perceived as legitimate. They missed two important governance aspects: communication and participation (Casiro and Regehr 2018; Kezar 2004). Consequently, they developed workarounds to recover their loss of freedom and made adjustments to the program without reporting it. They acknowledged the risks of fragmentation as well as curriculum bloating, with students ultimately suffering the consequences. Additionally, their circumventing behaviors resonate with the concept of ‘micropolitics’ in schools (Brosky 2011; Kelchtermans and Ballet 2002). Teachers have been known to use strategies and tactics to further their interests, which is reflected in their attempts to maintain their preferred work conditions, protecting them against changes and trying to recover them, if necessary (Kelchtermans and Ballet 2002). Awareness of such behavior seems vital in reaching the desired curriculum change goals.

More broadly, our study illustrates the importance of academic governance in medical schools. In order to align curricular practices with educational concepts and ideas, change leaders should emphasize the importance of defining and explicating governance procedures. The illustrative example of Institute 2 shows what will happen when this is not the case. Here, the experienced lack of clarity about decision making, leadership, responsibilities and tasks, in combination with an ill-defined educational concept, created “implementation chaos” with educators ending up making their own interpretations of what was expected. Finally, a variation of methods and translations of the educational concept were used in practice. Although researchers emphasized not to consider adjustment of procedures and structures the best solution for improving malfunctioning governance processes (Kezar 2004), our study showed that such activities also should not be underestimated. In line with others (Birnbaum 2004), we believe that ‘hard’ governance procedures and structures function as foundations of governance systems, together with ‘soft’, informal, social powers that are negotiated in interactions and relationships among stakeholders within the same system. Scholars emphasized the importance of understanding the role of governance within an organization, matching it with its local environmental, historical and cultural context (Guzick and Wilson 2018; Campbell and Bray 2018; Stensaker and Vabø 2013; Birnbaum 2004). Various governance structures might work well, however, as with many organizational efforts, it heavily depends on the culture and people involved (Guzick and Wilson 2018; Birnbaum 2004). Our study empirically supports these previous observations in medical educational curriculum enactment.

### Strengths and limitations

One of the strengths of our study was the use of a relatively new method in medical education: rich pictures. The rich picture method enabled us to capture the complexity of governance processes in curriculum change in one picture. Participants were able to explore the entire process, covering all elements that mattered to them. Rich pictures helped us map a complex story of multiple interacting factors and actors, a story that could have been lost when told in a linear prose style (Checkland 2000). Both a strength and limitation of our study approach was the recruitment of participants. We left the selection (partly) to the local curriculum leaders. They knew their context better than we did and could help us targeting the participants. On the other hand, we therefore did not have full control of whom we connected with, as this depended on who was invited and who chose to participate. Furthermore, for feasibility reasons, for each institute we had planned three fixed interviews days, which might have caused a selection bias due to availability issues. The fact that the deans were asked to invite potential participants may have caused even more selection bias, because they could only have invited their preferred educators. However, we had the impression that there was a good balance of people who were able to be critical and people who appreciated aspects of the governance processes. Possibly, participants might have felt obliged to participate in our study since the request came from the dean. Reflecting on the emails we received, we felt that people were truly willing to join in the research. Also during the interviews, participants expressed their appreciation for this chance to reflect on the process, which in many cases was not addressed in their institutes, which they regretted in hindsight. Another limitation of our study design was that we asked participants to look back at a past process, which could have created a recall bias. However, this could also be used as an advantage, since our design allowed educators to reflect on a past experience, which allows, over time, critically considering what was of real importance, and what not.

Building on that, future studies could follow governance processes throughout an entire curriculum change project, to better understand their course and critical success factors for different stages of the process. Additionally, we only considered governance from the perspective of middle management educators who had a teaching as well as a coordinating role. This provided us with a rather unique view of the complex process of curriculum change. Future research, however, could cover other stakeholder perspectives to further enrich our understanding of the role of governance, for example, perspectives of teachers without coordinating positions, board members, educational scientists or students. Finally, our study was conducted in three Dutch medical schools and showed the highly context-relatedness of governance and the importance of project alignment with local contexts. To advance scholarly debates and discourse on this topic and improve our understanding of how to support educators operating within undergraduate medical curricula, we recommend replication of our research in other educational systems, worldwide.

## Conclusion

Acknowledging the role of governance in curriculum change processes is crucial for anyone who needs to plan, implement or monitor curriculum change projects. Our paper highlights the importance of paying more attention to governance in making strategies work at the critical level of translating ideas—from paper to people—in medical school practice. To advance curriculum change processes and improve their desired outcomes it seems important to define, explicate and balance both hard and soft governance processes.

## Footnotes

1 Raamplan 2009 (Framework 2009, also known as ‘the Dutch Blueprint’) describes the attainment targets to which medical programs in the Netherlands have to align.
